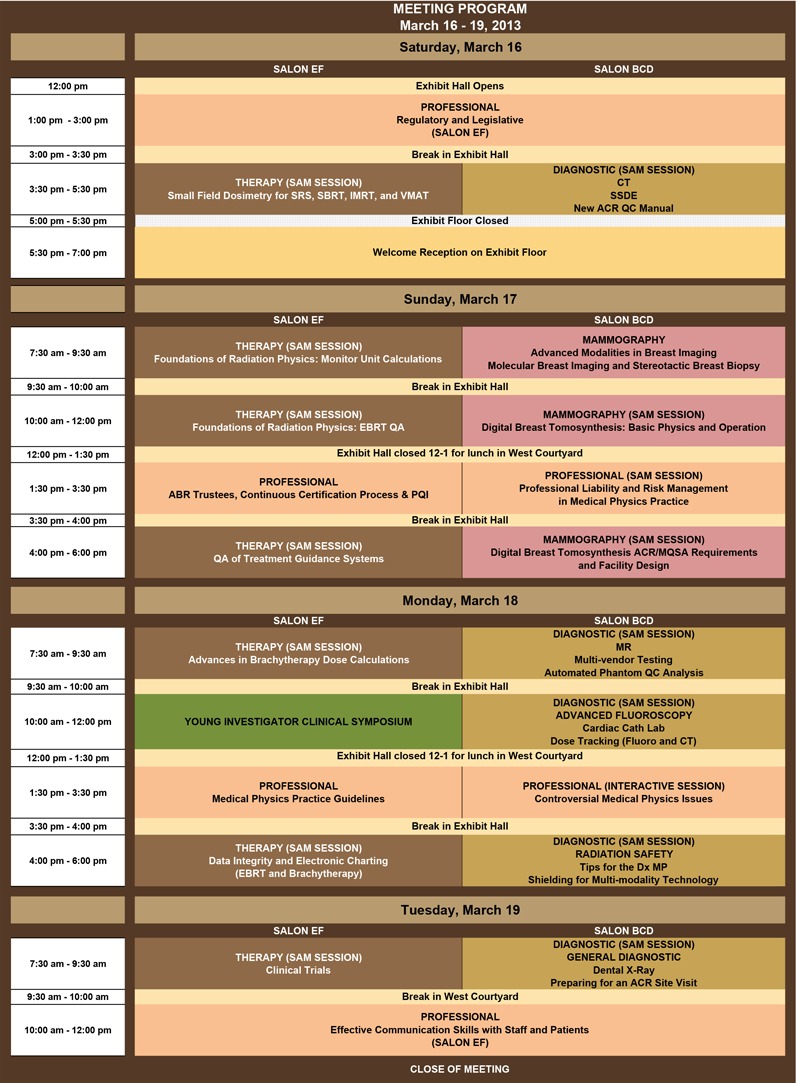# MEETING PROGRAM

**DOI:** 10.1120/jacmp.v14i3.4459

**Published:** 2013-05-06

***Available on‐line at*** www.aapm.org/meetings/2013SCM/

**2013 AAPM Spring Clinical Meeting**

**March 16 – 19, 2013**

**Phoenix, AZ**

**Chair**

David E. Hintenlang, PhD

University of Florida

Gainesville, FL

**Organizers**

**THERAPY TRACK**

Joann I. Prisciandaro, PhD

Radiation Oncology

University of Michigan

Ann Arbor, MI

Dimitris Mihailidis, PhD

Rad Onc and Med Phys

Charleston Radiation Therapy Cons

Charleston, WV

**PROFESSIONAL TRACK**

David E. Hintenlang, PhD

University of Florida

Gainesville, FL

**DIAGNOSTIC TRACK**

Robert A. Pooley, PhD

Radiology

Mayo Clinic

Jacksonville, FL

**YOUNG INVESTIGATOR PROGRAM**

Jessica B. Clements, MS

Medical Physics

Texas Health Presbyterian Hospital Dallas

Dallas, TX

Brian Wang, PhD

University Utah

Huntsman Cancer Hospital

Salt Lake City, UT

Jean M. Moran, PhD

Dept of Radiation Oncology B2C438

Ann Arbor, MI

Jessica B. Clements, MS

Medical Physics

Texas Health Presbyterian Hospital Dallas

Dallas, TX

**MAMMOGRAPHY TRACK**

William Geiser, MS

Imaging Physics

M.D. Anderson Cancer Center

Houston, TX

**Figure 1:**